# Effects of Low-Frequency Deep Brain Stimulation in Bilateral Zona Incerta for a Patient With Tremor and Cerebellar Ataxia

**DOI:** 10.5334/tohm.925

**Published:** 2024-08-21

**Authors:** Ami Kumar, Kristen L. Matulis, Zena A. Fadel, Alexander S. Fanning, Christian J. Amlang, Sheng-Han Kuo

**Affiliations:** 1 Department of Neurology, Columbia University Irving Medical Center and the New York Presbyterian Hospital, New York, USA; 2 Initiative for Columbia Ataxia and Tremor, Columbia University Irving Medical Center, New York, NY, USA; 3 Teachers College, Columbia University, New York, NY, USA; 4 Department of Neurology, Columbia University Irving Medical Center and the New York Presbyterian Hospital, New York, NY, USA; 5 Department of Neurology, SUNY Downstate, Brooklyn, NY, USA

**Keywords:** DBS, low-frequency stimulation, caudal zona incerta, cerebellar ataxia

## Abstract

**Background:**

Whether low-frequency deep brain stimulation (DBS) in the caudal zona incerta (cZi) can improve cerebellar ataxia symptoms remains unexplored.

**Case Report:**

We report a 66-year-old man initially diagnosed with essential tremor and subsequently developed cerebellar ataxia after bilateral cZi DBS implantation. We tested the effects of low-frequency DBS stimulations (sham, 10 Hz, 15 Hz, 30 Hz) on ataxia severity.

**Discussion:**

Low-frequency cZi DBS improves ataxic speech at 30 Hz, but not at 10 Hz or 15 Hz in this patient. Low-frequency DBS did not improve gait or stance. Therefore, low-frequency stimulation may play a role in treating ataxic speech.

**Highlights:**

The finding of this case study suggests that bilateral low-frequency DBS at 30 Hz in the caudal zona incerta has the potential to improve ataxic speech but has limited impact on gait and stance. The involvement of zona incerta in speech warrants further investigation.

## Introduction

Ataxia is a classical clinical symptom originating from cerebellar dysfunction that profoundly impacts the precision of motor control. It manifests as a lack of coordination and balance, leading to unsteady movements, slurred speech, and difficulties with posture and gait [1,2]. While deep brain stimulation (DBS) targeting the ventral intermediate nucleus (Vim) of the thalamus, which is situated downstream of the cerebellum, is highly effective in treating tremor [3,4], it is not effective in treating cerebellar ataxia. This is clearly demonstrated in several reports of patients with spinocerebellar ataxias that show thalamic DBS improves tremor but not ataxia [5,6,7,8]. The reason why thalamic DBS is effective to treat tremor but not ataxia remains a mystery, which suggests that 1) tremor and ataxia have distinct pathophysiology, 2) traditional high-frequency stimulation (~130 Hz) is effective in disrupting tremor, but not effective in improving ataxia.

Thalamic DBS is effective in treating tremor, but recent studies have shown that the caudal Zona incerta (cZi) has emerged as a promising target for DBS to treat tremor [9,10,11]. Moreover, high-frequency deep brain stimulation (DBS) of the cZi, has been shown to improve speech in essential tremor patients suffering from vocal tremor [12]. The cZi receives ample innervation from the cerebellum with minor projections back to it. In addition, cZi also projects to the basal ganglia, brainstem, and spinal cord [13,14,15,16,17,18]. Whether cZi DBS can improve cerebellar ataxia, to the best of our knowledge, has not yet been determined.

Another important consideration for DBS is stimulation parameters. Recent animal studies have suggested that low-frequency stimulation can improve cerebellar ataxia. Low-frequency DBS at 30 Hz, targeting the deep cerebellar nuclei (DCN), improved ataxia-like locomotion in *Shaker* rats [19], while another study showed DCN DBS at 13 Hz ameliorated ataxia-like locomotion in *Car8* mice [20]. These studies collectively demonstrated the early promise of low-frequency DBS of the DCN for improving cerebellar ataxia in animal models, indicating that low-frequency stimulation can potentially normalize ataxia-related pathophysiology.

Given the fact cZi receives strong innervation from the DCN, we hypothesized that low-frequency DBS in cZi may be effective in reducing ataxia symptoms. We tested this hypothesis in a unique patient who was initially diagnosed with essential tremor and subsequently received bilateral cZi DBS implants. Unfortunately, the patient developed profound cerebellar ataxia after the surgery, providing an opportunity to test the effects of bilateral cZi low-frequency stimulation for cerebellar ataxia.

## Case Description

### Prior DBS history

A 66-year-old man developed progressive tremor in his hands at the age of 40. At age 50, he was unable to write or perform activities of daily living, such as eating and drinking water, due to tremor. His tremor was partially responsive to clonazepam, but he was still disabled from his hand tremor. His brain magnetic resonance imaging (MRI) demonstrated no cerebellar atrophy. He was diagnosed with essential tremor. He underwent staged bilateral cZi DBS surgery at the age of 64, with a three-month interval between the placements of the left and right cZi DBS leads. He had no complications during the DBS surgeries. His post-operative computed tomography confirmed the lead placements in bilateral cZi. However, he developed profound unsteady gait and slurred speech, symptoms of cerebellar ataxia, after the second stage of the cZi DBS surgery, before the DBS was programmed. During initial DBS programming at the frequencies of 130–185Hz, his tremor improved but his cerebellar ataxia remained. To exclude the possibility that cerebellar ataxia was produced by the prelemniscal fiber stimulations, his DBS was subsequently turned off, but his cerebellar ataxia persisted to the degree that he could no longer walk independently, and his speech became difficult to understand.

### Low-Frequency DBS investigation

He then came to Columbia University Ataxia Center for a second opinion. On exam, he had severe gait ataxia, and he could not walk without assistance. His hand movements were impaired by a combination of tremor and dysmetria, a sign of cerebellar ataxia. He had impaired finger chase, finger-to-nose test, fast alternating movements, and heel-to-shin test. His Scale for Assessing and Rating Ataxia (SARA) was 21.5 at baseline measurement. Genetic analyses showed a mutation in m.8344 A>G *MT-TK* mitochondrial gene with 85% heteroplasmy, confirming a diagnosis of myoclonic epilepsy with ragged-red fibers (MERRF) [21]. MERRF is a rare mitochondrial disease, that can present at any age, with primary symptoms involving myoclonus, seizures, cerebellar ataxia, myopathy, and ragged red fibers on muscle biopsy. Other symptoms that can occur in MERRF include dementia, optic atrophy, bilateral deafness, peripheral neuropathy, and spasticity, which fortunately our patient does not have [21].

Given that his ataxia was refractory to either pharmacology and/or high-frequency cZi DBS, and could possibly be due to his underlying condition of MERRF, prior literature suggests the benefits of low-frequency DBS in reducing ataxia-like symptoms in animals [19,20], therefore, we conducted a single day, single-blinded trial of low-frequency cZi DBS in this patient. We assessed ataxia severity with clinical ratings of SARA [22], used APDM mobility lab wearable sensors to detect postural sways and used an accelerometer to assess hand tremor [23]. We assessed the speech clarity using the speech analysis software, Praat (version 6.4.12), [24] which has been extensively validated in patients with cerebellar ataxia [25,26,27,28]. Praat measures speech clarity quantitatively using Acoustic Voice Quality Index (AVQI), with a higher AVQI demonstrating worsening of speech [29].

**Figure 1:**
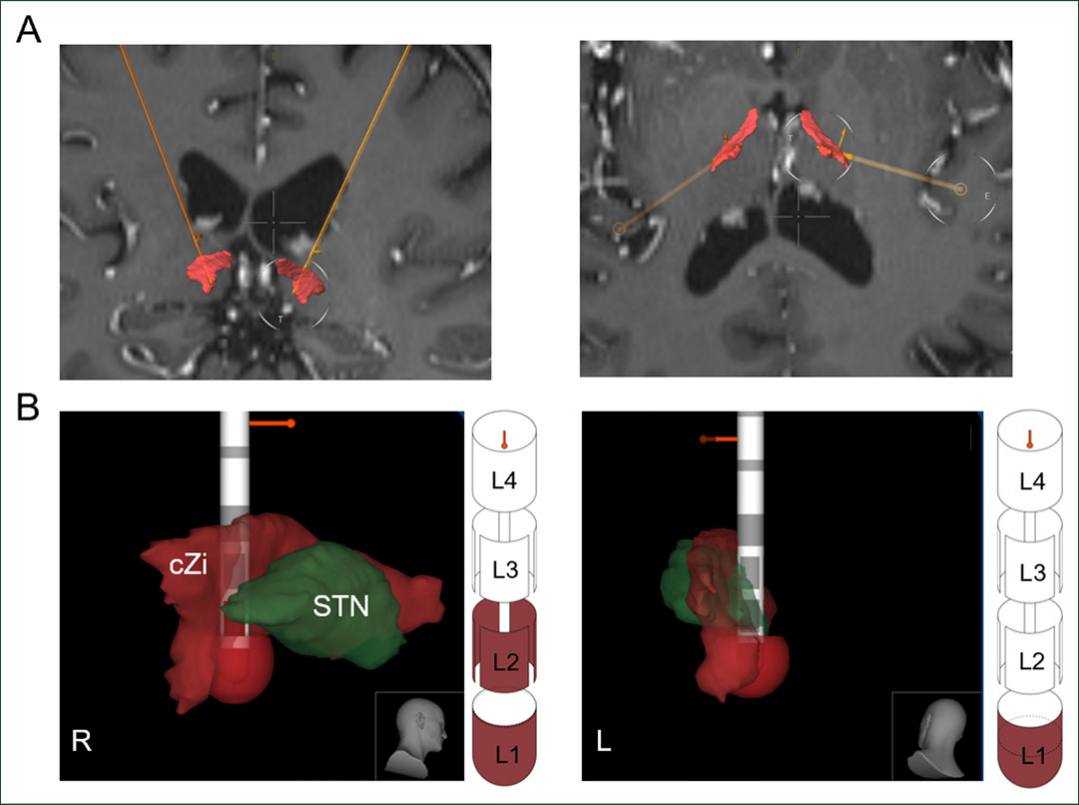
**Reconstruction of DBS electrodes and programming. (A)** Coronal and axial sections of the patient’s MRI overlayed with the placement of the right and left DBS leads in the cZi, indicated as the red region. **(B)** Programming of the leads targeting the cZi, with monopolar activation of the right lead consisting of 70% L1 and 30% L2 ring mode contact, and the left lead consisting of 100% L1 contact. cZi caudal Zona incerta, STN subthalamic nucleus.

We first obtained the baseline assessments (Table 1), and then tested four DBS settings (sham, 10 Hz, 15 Hz, and 30 Hz). These frequencies were selected based on prior literature suggesting the benefits of low-frequency DBS in reducing ataxia-like motor deficits in animal models at 13 Hz and 30 Hz [19,20]. Since frequencies can be selected in increments of 5, we chose 10 Hz and 15 Hz as they are closest to 13 Hz, and additionally selected 30 Hz as a frequency of interest. We did not include any high-frequency stimulation in this trial due to these settings leading to worsening of cerebellar ataxia in prior programming sessions in the patient, before coming to our center. A nurse practitioner specializing in DBS (KM) adjusted the DBS settings, whereas a scientist specializing in wearable sensors (AK) assessed the wearable device measures. The clinical assessments of the patient were performed by a movement disorders neurologist (CA) using video-taped movement disorders examination. The patient, and the movement disorders neurologist (CA) were blinded and were not aware of the DBS settings. DBS contacts were chosen based on the modeling from the co-registration of post-operative MRI using BrainLab imaging software to precisely target the cZi (Figure 1A). The left lead consisted of 100% monopolar activation at contact L1, and the right lead consisted of 70% monopolar activation at contact L1, with 30% activation in ring mode at contact L2 (Figure 1B). In all conditions the intensity was set at 1 mA, with pulse width as 60µs to specifically test the effects of frequency on clinical symptoms. The patient’s history indicates that setting of higher amplitudes above 1.5 mA was not well-tolerated, causing dizziness and lightheadedness. Therefore, to standardize programming, we opted to keep these settings consistent across all conditions. The duration of DBS ON for each stimulation frequency ranged between 13–15 minutes and all the assessments were performed during DBS ON to study the real time effects. The patient rested for 15 minutes between different DBS settings.

**Table 1:** SARA assessments for DBS stimulation conditions.


SARA ASSESSMENTS	BASELINE	SHAM	10 HZ	15 HZ	30 HZ	SCORE RANGE

Gait	6	6	6	6	6	0–8

Stance	2	2	2	2	2	0–6

Sitting	2	2	2	2	2	0–4

Speech	3	3	3	3	1	0–6

Finger chase – right	1	1	1	2	1	0–4

Finger chase – left	2	2	2	2	2	0–4

Nose Finger – right	2	2	2	3	2	0–4

Nose Finger – left	3	3	3	3	3	0–4

Fast alternating movement – right	2	2	2	1	1	0–4

Fast alternating movement – left	2	2	2	2	2	0–4

Heel shin slides – right	2	2	2	2	1	0–4

Heel shin slides – left	3	3	2	3	2	0–4

Total	21.5	21.5	21	22	18	


**Figure 2:**
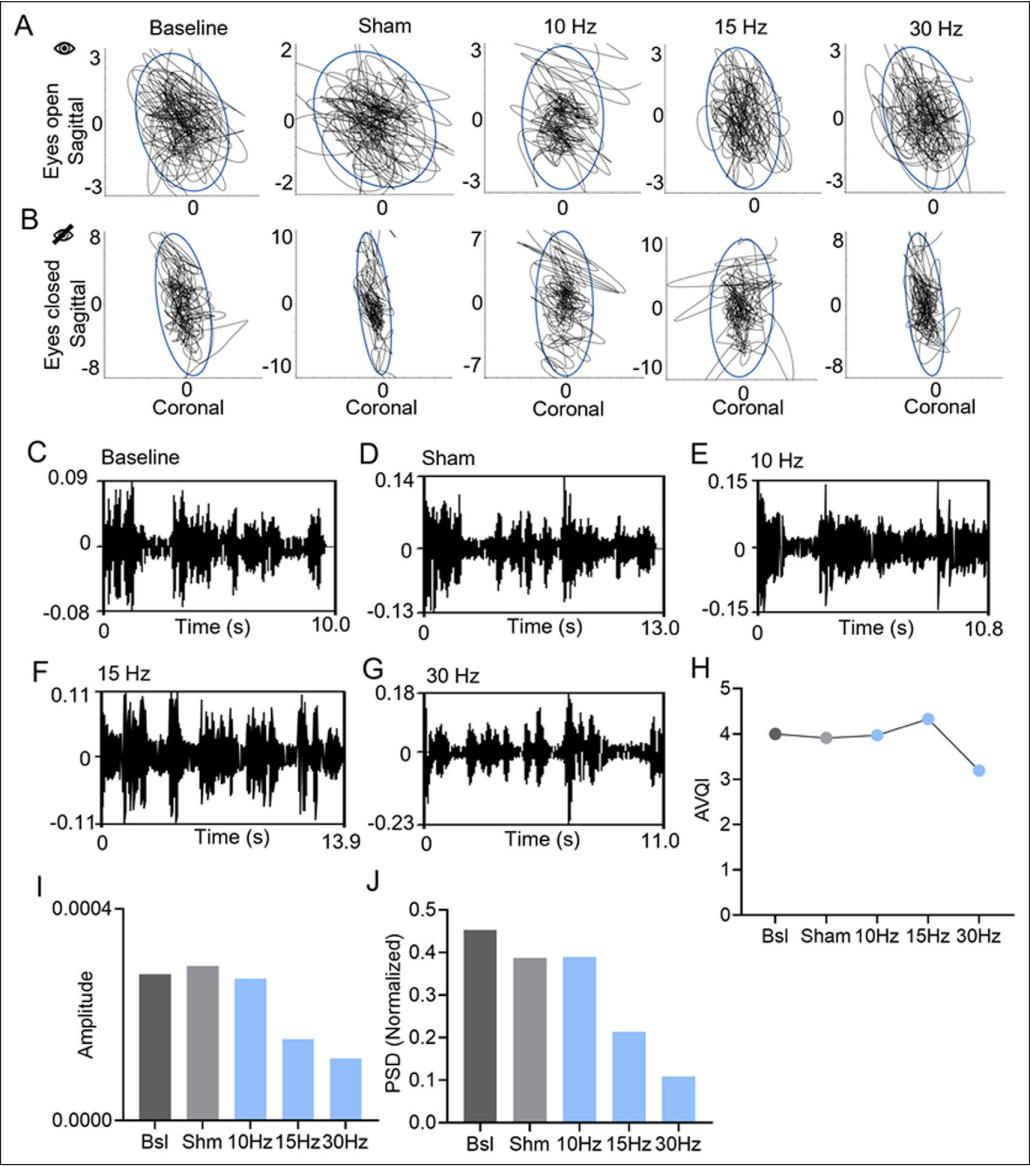
**Ataxia and tremor assessments. (A)** Postural sway area maps using APDM sensors with eyes open and feet apart for all conditions, demonstrating no drastic changes with the low-frequency stimulation as compared to baseline. **(B)** Similarly, with eyes closed and feet apart condition, no clear differences in sway measures were observed for the low- frequency stimulation conditions. **(C-G)** Speech audio spectrum for baseline, sham, 10 Hz, 15 Hz and 30 Hz stimulation conditions. **(H)** Acoustic voice quality index (AVQI) for all DBS stimulation conditions, depicting a reduction in AVQI for 30 Hz stimulation and showing an improvement in speech compared to other conditions. **(I)** A reduction in tremor amplitude was observed for the 30 Hz condition, as compared to baseline, sham and other low-frequency stimulation conditions. **(J)** Power spectral density (PSD) analysis also showed a reduction in spectral power at 30 Hz stimulation, indicating tremor reduction. Bsl baseline, Shm sham.

We did not observe adverse effects in any of the stimulation conditions. While sham stimulation did not demonstrate placebo responses, we found that stimulation at 10 Hz and 15 Hz did not worsen or improve gait or stance ataxia (Table 1). Consistently, we did not observe any effects of different low-frequency stimulation in postural sway measurements with eyes-open and eyes-closed while the patient stood on a firm surface (Figure 2A, B). Interestingly, we observed cZi DBS at 30 Hz provided a modest improvement in speech (SARA sub-score: baseline: 3, sham: 3, 10 Hz: 3, 15 Hz: 3 and 30 Hz: 1). The patient and his family also noticed discernable differences in his speech at 30 Hz stimulation but not sham stimulation or other frequency stimulations (**Supplementary files 1–3**). Consistently, his speech analysis showed an improvement of AVQI with 30 Hz stimulation when compared to other stimulation conditions (AVQI scores: baseline: 4.00, sham: 3.91, 10 Hz: 3.97, 15 Hz: 4. 33, and 30 Hz: 3.19; lower number indicating a better speech clarity) (Figure 2C–H). Further, a mild improvement in limb ataxia was seen during fast-alternating hand movements (baseline: 2, sham: 2, 10 Hz: 2, 15 Hz: 1.5, and 30 Hz: 1.5) and heel-shin slides (baseline: 2.5, sham: 2.5, 10 Hz: 2, 15 Hz: 2.5, and 30 Hz: 1.5). Interestingly, we observed a reduction in hand tremor amplitude and normalized power spectral density for the 30 Hz stimulation condition as compared to baseline, sham and other low-frequency stimulation conditions (Figure 2I, J).

## Discussion

In this study we report a case of low-frequency DBS to bilateral cZi in a patient initially diagnosed with essential tremor, who developed worsening of cerebellar ataxia after DBS surgeries. We found 30 Hz stimulation, but not 10 Hz or 15 Hz, modestly improved ataxic speech. Additionally, 30 Hz stimulation showed benefits in reducing tremor. Further exploration is needed to elucidate the role of cZi in the brain circuit linked to speech.

Speech is the primary means to communicate with each other, and slurred speech is one of the most bothersome symptoms that people with cerebellar ataxia experience. However, we currently do not have the means to improve speech in individuals with cerebellar ataxia. Our study suggested that low-frequency bilateral cZi DBS might improve ataxic speech.

Previous animal model studies have demonstrated that DBS at 30 Hz, targeting the deep cerebellar nuclei, improved ataxia-like locomotion in *Shaker rats*. This study suggests that while high-frequency DBS acts as an informational lesion or functional deafferentation, low-frequency DBS (<50 Hz) on the other hand might enhance network output, improving the transmission of coordination-relevant information through cerebellar pathways, that may include the dentate, pontine mossy fibers, and olivary climbing fiber collaterals [19], which prompted us to test the low frequency stimulations on cerebellar ataxia.

The cZi has widespread connectivity with much of the brain [13,18]. While it is possible that cZi DBS could modulate speech via its cerebello-cortical connections, cZi also has distinct connections to basal ganglia, basal pontine nuclei and pedunculopontine tegmental nucleus, [15,16], which can be alternative pathways to affect speech function. cZi receives somatotopic input from cerebral cortex with the head and neck having the largest representation, coupled with the dense connectivity with brainstem structures, suggesting alternative pathways of regulating the motor function of muscles controlling speech production.

Given this, it is plausible that ataxia and tremor modulation by cZi stimulation might involve these cerebellar projections. Further exploration of low-frequency cZi DBS as a target for ataxic speech should be considered in future therapeutic development. While slurred speech is a known potential adverse effect of bilateral VIM DBS in patients with essential tremor and Parkinson’s disease, cZi DBS appears to have less speech-related adverse effects [30]. Our study suggests that cZi DBS could be further explored as a potential target for low-frequency neuromodulation for slurred speech.

Furthermore, given that the patient has MERRF, which includes ataxia as one of its symptoms, it is possible that stress-related to the brain surgery may have unmasked these underlying ataxia symptoms from energy failure related to mitochondrial dysfunction. Therefore, clinicians should be cautious when considering DBS surgery in patients with mitochondrial disorders.

As this was a pilot, single case study, the results should be interpreted cautiously, and several limitations should be considered. First, we only studied the acute effects, based on our understanding that cZi DBS has acute effects of tremor. Future studies should include different time points to fully determine if cZi DBS could have delayed effects on cerebellar ataxia. Second, our results are based on a single trial in each setting. To reliably demonstrate the effects of cerebellar ataxia, a larger study with multiple trials should be considered, as patients’ symptoms, measured by SARA scale, could have inherent variability. Additionally, we only tested the standard 60 µs pulse width without exploring other pulse width settings. Future studies should also include different combinations of frequencies and pulse widths for cZi DBS on cerebellar ataxia. In summary, our study suggests that bilateral low frequency cZi DBS at 30 Hz may improve speech in patients with cerebellar ataxia. This finding suggests potential avenues for future investigations into low-frequency stimulation as a viable therapeutic option for patients with cerebellar ataxia, paving the way for further testing and refinement of treatment approaches in this population.

## Supplementary files

Supplementary file 1. Audio recording at baseline.

Supplementary file 2. Audio recording at sham stimulation.

Supplementary file 3. Audio recording at 30 Hz stimulation.
